# Supplementary material for: Real-World Immunogenicity and Reactogenicity of Two Doses of Pfizer-BioNTech COVID-19 Vaccination in Children Aged 5–11 Years
Source: Vaccines (Basel). 2022 Nov 18;10(11):1954. doi: 10.3390/vaccines10111954 (PMC9693260; doi:10.3390/vaccines10111954)
Supplement: Supplementary file 1 [file vaccines-10-01954-s001.zip › vaccines-2035280-supplementary.pdf]

Supplements

Table S1. Questionnaire for data collection and adverse effects (carried out online a week after vaccination date).

	Question	Answer 1	Answer 2	Comments
1.	What is your child date of birth?			
2.	What is your child gender?	Male	Female	
3.	Did the child experience pain at the injection site?	Yes	No	
4.	Did the child experience redness at the injection site?	Yes	No	
5.	Did the child experience swelling at the injection site?	Yes	No	
6.	Did the child experience itching at the injection site?	Yes	No	
7.	Did the child experience other local symptoms?	Yes	No	Describe
8.	How many days did the child experience the local symptoms?	Number of days		
9.	Did your child experience a fever above 37.5°c? How many days did it last?	Yes	No	Number of days
10.	Did your child experience fatigue or weakness? How many days did it last?	Yes	No	Number of days
11.	Did your child experience muscle aches (myalgia)? How many days did it last?	Yes	No	Number of days
12.	Did your child experience enlargement of glands? How many days did it last?			Number of days
13.	Did your child experience a headache? How many days did it last?	Yes	No	Number of days
14.	Did your child experience facial nerve palsy? How many days did it last?	Yes	No	
15.	Did your child experience paresthesia? How many days did it last?	Yes	No	
16.	Did your child experience allergy? How many days did it last?	Yes	No	
17.	Did your child experience changes in laboratory tests, if performed?	Yes	No	
18.	Did your child miss school? For how many days?	Yes	No	Number of days
19.	Did your child need medical treatment?	Describe		Describe
20.	Did your child need hospitalization due to the vaccine side effects?	Yes	No	Describe

Table S2. Questionnaire of description of symptoms including long COVID-19. (Carried out online at day 180 of the study)

	Question	Answer 1	Answer 2	Comments
1.	Was your child infected with COVID-19?	Yes	No	
2.	How many days has your child been sick with COVID-19?	Number of days		
3.	How many school days did your child lose?	Number of days		
4.	Were there any symptoms?	Yes	No	
5.	Did your child experience a fever above 37.5 for up to 2 days?	Yes	No	
6.	Did your child experience a fever above 37.5 for over than 2 days?	Yes	No	
7.	Did your child experience fatigue or weakness?	Yes	No	
8.	Did your child experience a headache?	Yes	No	
10.	Did your child experience reduced/change sense of test and smell?	Yes	No	
11.	Did your child experience shortness of breath?	Yes	No	
12.	Did your child experience cough?	Yes	No	
13.	Did your child experience rhinorrhea?	Yes	No	
14.	Did your child experience a sore throat?	Yes	No	
15.	Did your child experience gastrointestinal inconvenience/diarrhea?	Yes	No	
16.	Did your child need hospitalization?	Yes	No	
17.	Did your child experience any other symptoms?	Describe		
	**Long COVID-19**			
1.	Did your child experience any long COVID-19 symptoms (more than 2 weeks after the initial illness?	Yes	No	
2.	Did your child experience long COVID-19 symptoms such fatigue?	Yes	No	
3.	Did your child experience long COVID-19 symptoms such as trouble with concentration/ Confusion/ Memory loss?	Yes	No	
4.	Did your child experience long COVID-19 symptoms such feelings of sadness/ depression?	Yes	No	
5.	Did your child experience long COVID-19 symptoms such as Agitation\ Anxiety?	Yes	No	
6.	Did your child experience long COVID-19 symptoms such as reduced/change sense of test and smell?	Yes	No	
7.	Did your child experience long COVID-19 symptoms such as shortness of breath?	Yes	No	
8.	Did your child experience long COVID-19 symptoms such as cough?	Yes	No	
9.	Did your child experience long COVID-19 symptoms such as headache?	Yes	No	
10.	Did your child experience long COVID-19 symptoms such as gastrointestinal inconvenience/diarrhea?	Yes	No	
11.	Did your child experience long COVID-19 symptoms such as decreased physical fitness?	Yes	No	
12.	Did your child experience long COVID-19 symptoms such as chest pain?	Yes	No	

13.	Did your child experience long COVID-19 symptoms such palpitations?	Yes	No
14.	Did your child experience long COVID-19 symptoms such as shortness of breath at rest?	Yes	No
15.	Did your child experience any other symptoms?	Describe	

Table S3. GeoMean (CI 95%) of IgG titer of all children and of infected and uninfected children (BAU/IU).

IgG	All Children GeoMean (CI95%)	Infected Children GeoMean (CI95%)	Uninfected Children GeoMean (CI95%)
**Day 0**	0.44 (0.36 -0.55)	-	-
**Day 21**	178.5 (129.2 – 245.3)	-	-
**Day 90**	1523.0 (1163.0 -1996.0)	1670.0 (1131.0 - 2466.0) (P>0.05)	1291.0 (929.6 - 1792.0)
**Day 180**	1076 (712.3 -1624.0)	1479.0 (878.2 - 2490.0) (*p<0.05)	535.3 (288.4 - 933.6)

Table S4. GeoMean (CI 95%) of neutralizing antibody titer of all children and of infected and uninfected children.

IgG	All Children GeoMean (CI95%)	Infected Children GeoMean (CI95%)	Uninfected Children GeoMean (CI95%)
**Day 0**	0.1 (0.1-0.12)	-	-
**Day 21**	78.2 (54.4 – 114.3)	-	-
**Day 90**	892.7 (630.5–1264.0)	1057.0 (645.9 - 1730.0) (P>0.05)	664.0 (425.6 - 1036.0)
**Day 180**	1203.0 (698.5–2072.0)	2250.0 (1185.0 – 4272.0) (*p<0.05)	658.8 (291.9 - 1487.0)

Table S5. Description of long COVID-19 symptoms.

	Long COVID-19 symptoms	N (%)
1.	Any long COVID-19 symptoms (more than 2 weeks after the initial illness)	3
2.	Fatigue/weakness	1
3.	Trouble with concentration/Confusion/Memory loss	0
4.	Sadness/depression	0
5.	Agitation\Anxiety	0
6.	Reduced/change sense of taste and smell	1
7.	Shortness of breath	0
8.	Cough	0
9.	Headache	1
10.	Gastrointestinal inconvenience/diarrhea	0
11.	Decreased physical fitness	0
12.	Chest pain	0
13.	Palpitations	0
14.	Shortness of breath at rest	0
15.	Any other symptoms	0
